# Progress Toward Polio Eradication — Worldwide, January 2020–April 2022

**DOI:** 10.15585/mmwr.mm7119a2

**Published:** 2022-05-13

**Authors:** Audrey Rachlin, Jaymin C. Patel, Cara C. Burns, Jaume Jorba, Graham Tallis, Aidan O’Leary, Steven G.F. Wassilak, John F. Vertefeuille

**Affiliations:** ^1^Epidemic Intelligence Service, CDC; ^2^Global Immunization Division, Center for Global Health, CDC; ^3^Division of Viral Diseases, National Center for Immunization and Respiratory Diseases, CDC; ^4^Polio Eradication Department, World Health Organization, Geneva, Switzerland.

In 1988, the World Health Assembly established the Global Polio Eradication Initiative (GPEI). Since then, wild poliovirus (WPV) cases have decreased approximately 99.99%, and WPV types 2 and 3 have been declared eradicated. Only Afghanistan and Pakistan have never interrupted WPV type 1 (WPV1) transmission. This report describes global progress toward polio eradication during January 1, 2020–April 30, 2022, and updates previous reports (*1*,*2*). This activity was reviewed by CDC and was conducted consistent with applicable federal law and CDC policy.* Five WPV1 cases were reported from Afghanistan and Pakistan in 2021, compared with 140 in 2020. In 2022 (as of May 5), three WPV1 cases had been reported: one from Afghanistan and two from Pakistan. WPV1 genetically linked to virus circulating in Pakistan was identified in Malawi in a child with paralysis onset in November 2021. Circulating vaccine-derived polioviruses (cVDPVs), with neurovirulence and transmissibility similar to that of WPV, emerge in populations with low immunity following prolonged circulation of Sabin strain oral poliovirus vaccine (OPV) (*3*). During January 2020–April 30, 2022, a total of 1,856 paralytic cVDPV cases were reported globally: 1,113 in 2020 and 688 in 2021, including cases in Afghanistan and Pakistan. In 2022 (as of May 5), 55 cVDPV cases had been reported. Intensified programmatic actions leading to more effective outbreak responses are needed to stop cVDPV transmission. The 2022–2026 GPEI Strategic Plan objective of ending WPV1 transmission by the end of 2023 is attainable (*4*). However, the risk for children being paralyzed by polio remains until all polioviruses, including WPV and cVDPV, are eradicated. 

## Poliovirus Vaccination

Since the 2016 withdrawal of Sabin polio vaccine virus type 2 and the globally synchronized switch from trivalent OPV (tOPV, including Sabin types 1, 2, and 3) to bivalent OPV (bOPV, including Sabin types 1 and 3) in all OPV-using countries, bOPV and injectable inactivated poliovirus vaccine (IPV) (including all three serotypes) have been used in routine immunization programs worldwide. cVDPV type 2 (cVDPV2) has been the predominant cause of cVDPV outbreaks since 2006 and informed the rationale for the switch to bOPV. Monovalent OPV Sabin type 2 (mOPV2) is reserved for cVDPV2 outbreak response campaigns (*3*).

In 2020,^†^ the estimated global coverage with ≥3 doses of oral or inactivated poliovirus vaccine (Pol3) in infants aged ≤1 year received during routine childhood immunization (essential health services) was 83%, with 80% of children receiving ≥1 full dose or 2 fractional doses^§^ of IPV (IPV1). In Afghanistan, the national estimates of coverage with Pol3 and IPV1 were 75% and 65%, respectively, and in Pakistan, were 83% and 85%, respectively (*5*); however, coverage estimates at many subnational levels were considerably lower.

In 2020, GPEI supported the administration of approximately 665 million bOPV, 6 million IPV, 4 million monovalent OPV type 1 (mOPV1), 201 million mOPV2, and 51 million tOPV doses through 145 supplementary immunization activities (SIAs)^¶^ in 30 countries. For Afghanistan and Pakistan, both of which have simultaneous circulation of WPV1 and cVDPV2, GPEI approved the release of tOPV stocks to interrupt the transmission of both virus types. In 2021, approximately 726 million bOPV, 17 million IPV, 628 million mOPV2, and 51 million tOPV doses were distributed to 30 countries for use during 94 SIAs.

In November 2020, the World Health Organization (WHO) granted Emergency Use Listing** for novel OPV2 (nOPV2), designed to be more genetically stable than the Sabin strain and less likely to revert to neurovirulence (*6*). nOPV2 was first used during outbreak response SIAs in March 2021. Since that time, approximately 525 million nOPV2 doses have been released for use in 21 countries (as of May 5, 2022).

## Poliovirus Surveillance

The primary system for detecting poliovirus is case-based syndromic surveillance for acute flaccid paralysis (AFP), with confirmation by stool specimen testing done at one of 146 WHO-accredited laboratories across 92 countries, comprising the Global Polio Laboratory Network. The two primary indicators used to assess surveillance performance include the nonpolio AFP (NPAFP) rate^††^ and adequacy of collected stool specimens.^§§^ AFP surveillance indicators for 43 priority countries^¶¶^ experiencing or at high risk for poliovirus transmission were reported for 2020–2021 (*7*). Among the 43 priority countries, 32 (74%) met both surveillance indicator targets nationally in 2021. Subnational performance was highly variable (*7*).

Whether or not AFP surveillance performance indicator targets are met, gaps often exist in poliovirus detection subnationally. These gaps can be addressed through environmental surveillance (ES), the systematic collection and testing of sewage samples for poliovirus (*7*). In 2021, the total number of ES samples collected in countries with reported poliovirus circulation was 8,878 samples in 35 countries compared with 5,756 samples in 28 countries in 2020 (Table 1).

**TABLE 1:** Number and proportion of sewage samples with circulating wild polioviruses and circulating vaccine-derived polioviruses in environmental surveillance — worldwide, January 1, 2020–April 30, 2022*

Country	Jan 1–Dec 31, 2020		Jan 1–Dec 31, 2021		Jan 1–Apr 30, 2021		Jan 1–Apr 30, 2022	
	No. of samples	No. (%) with isolates	No. of samples	No. (%) with isolates	No. of samples	No. (%) with isolates	No. of samples	No. (%) with isolates
**Countries with reported WPV1-positive samples (no. and % of isolates refer to WPV1)**								
Afghanistan	418	35 (8)	474	1 (0)	153	1 (1)	151	0 (—)
Pakistan	830	434 (52)	851	65 (8)	284	55 (19)	253	1 (0)
**Countries with reported cVDPV-positive samples (cVDPV type) (no. and % of isolates refer to cVDPVs)**								
Afghanistan (2)	418	175 (42)	474	40 (9)	153	39 (26)	151	0 (—)
Benin (2)	70	5 (7)	143	1 (1)	31	1 (3)	20	0 (—)
Cameroon (2)	273	9 (3)	368	1 (0)	116	0 (—)	81	0 (—)
Central African Republic (2)	88	2 (2)	138	1 (1)	28	0 (—)	29	0 (—)
Chad (2)	77	3 (4)	64	1 (2)	17	0 (—)	13	0 (—)
China (3)	0	0 (—)	2	1 (50)	1	1 (100)	0	0 (—)
Côte d'Ivoire (2)	130	95 (77)	85	0 (—)	28	0 (—)	31	2 (6)
Democratic Republic of the Congo (2)	170	1 (1)	447	3 (1)	92	0 (—)	78	0 (—)
Djibouti (2)	0	0 (—)	71	5 (7)	0	0 (—)	10	0 (—)
Egypt (2)	557	1 (0)	916	12 (1)	228	9 (4)	205	2 (1)
Ethiopia (2)	51	4 (8)	32	0 (—)	9	0 (—)	9	0 (—)
Gambia (2)	0	0 (—)	39	9 (23)	0	0 (—)	11	0 (—)
Ghana (2)	184	20 (11)	189	0 (—)	68	0 (—)	40	0 (—)
Guinea (2)	67	1 (1)	143	2 (1)	42	0 (—)	2	0 (—)
Iran (2)	43	3 (7)	71	1 (1)	15	1 (10)	15	0 (—)
Israel (3)	2	1 (50)	9	5 (55)	0	0 (—)	25	25 (100)
Kenya (2)	170	1 (1)	176	1 (1)	59	1 (2)	39	0 (—)
Liberia (2)	34	7 (21)	86	14 (16)	27	12 (44)	14	0 (—)
Madagascar (1)	351	0 (—)	390	31 (8)	81	6 (7)	136	2 (1)
Malaysia (1,2)	201	14 (5)	122	0 (—)	49	0 (—)	11	0 (—)
Mali (2)	44	4 (9)	51	0 (—)	19	0 (—)	7	0 (—)
Mauritania (2)	0	0 (—)	72	7 (10)	0	0 (—)	22	0 (—)
Niger (2)	157	9 (6)	208	0 (—)	42	0 (—)	36	0 (—)
Nigeria (2)	1,294	5 (0)	2,427	300 (12)	541	6 (1)	755	29 (4)
Pakistan (2)	830	135 (16)	851	35 (4)	284	31 (11)	253	0 (—)
Palestinian Territory (3)	0	0 (—)	7	7 (100)	1	1 (100)	9	9 (100)
Philippines (2)	227	4 (2)	211	0 (—)	80	0 (—)	19	0 (—)
Republic of the Congo (2)	12	1 (8)	437	3 (1)	99	1 (1)	50	0 (—)
Senegal (2)	27	1 (4)	23	14 (61)	7	2 (29)	28	0 (—)
Sierra Leone (2)	0	0 (—)	208	9 (4)	60	8 (13)	44	0 (—)
Somalia (2)	87	26 (30)	134	1 (1)	37	0 (—)	41	0 (—)
South Sudan (2)	84	6 (7)	83	0 (—)	32	0 (—)	1	0 (—)
Sudan (2)	50	14 (28)	90	0 (—)	20	0 (—)	25	0 (—)
Tajikistan (2)	0	0 (—)	18	17 (94)	12	11 (92)	0	0 (—)
Uganda (2)	58	0 (—)	93	2 (2)	24	0 (—)	28	0 (—)
**Total**	**5,756**	**1,016 (14.5)**	**8,878**	**589 (6.6)**	**2,302**	**197 (8.6)**	**2,238**	**68 (3)**

**Abbreviations:** cVDPV = circulating vaccine-derived poliovirus; WPV1 = wild poliovirus type 1.

* Data as of May 5, 2022.

## Reported Poliovirus Cases and Isolations

**Countries reporting WPV cases and isolations.** In 2021, five WPV1 cases were reported from the two remaining countries with endemic polio: four from Afghanistan and one from Pakistan (Figure) (Table 2). The four WPV1 cases from two provinces in Afghanistan represent an 82% decrease from the 56 cases in 14 provinces reported in 2020. The single case reported from Balochistan province in Pakistan in 2021 represents a 99% decrease from 84 WPV1 cases in five provinces during 2020 (*8*). In 2022 to date, one WPV1 case has been reported in Afghanistan, from Paktika province on the eastern border near Pakistan, with paralysis onset on January 14. In Pakistan, two WPV1 cases have been reported in 2022, both from North Waziristan in Khyber Pakhtunkhwa province, with paralysis onset on April 9 and April 14.*** ES surveillance in Afghanistan detected one WPV1-positive sample from 474 (0.2%) in 2021, a 97% decrease from the 35 (8%) of 418 samples collected during 2020 (Table 1). The most recent positive sample was collected on February 23, 2021. In Pakistan in 2021, 65 WPV1 isolates were detected from 851 (8%) sewage samples, a 44% decrease from the same period in 2020 when 52% (434 of 830) of samples were WPV1-positive. A recently reported WPV1-positive ES sample was collected on April 5, 2022, in Khyber Pakhtunkhwa province.

**FIGURE:**
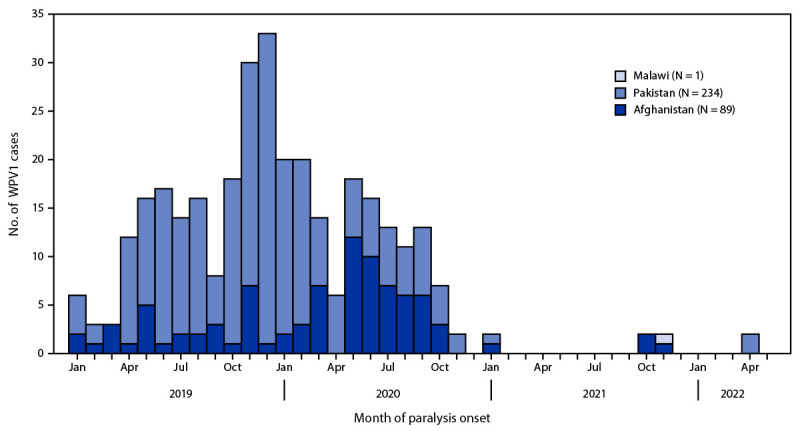
Number of wild poliovirus type 1 cases, by country and month of paralysis onset — worldwide, January 2020–April 2022* **Abbreviation:** WPV1 = wild poliovirus type 1. * Data as of May 5, 2022.

**TABLE 2:** Number of poliovirus cases, by country — worldwide, January 1, 2020–April 30, 2022*

Country	Reporting period							
	2020		2021		Jan–Apr 2021		Jan–Apr 2022	
	WPV1	cVDPV	WPV1	cVDPV	WPV1	cVDPV	WPV1	cVDPV
**With reported WPV1 cases (cVDPV type)**								
Afghanistan (2)	56	308	4	43	1	40	1	0
Malawi	0	0	1	0	0	0	0	0
Pakistan (2)	84	135	1	8	1	8	2	0
**With reported cVDPV cases (cVDPV type)**								
Angola (2)	0	3	0	0	0	0	0	0
Benin (2)	0	3	0	3	0	2	0	0
Burkina Faso (2)	0	65	0	2	0	1	0	0
Cameroon (2)	0	7	0	3	0	0	0	0
Central African Republic (2)	0	4	0	0	0	0	0	0
Chad (2)	0	101	0	0	0	0	0	0
Côte d’Ivoire (2)	0	64	0	0	0	0	0	0
Democratic Republic of the Congo (2)	0	81	0	28	0	10	0	26
Ethiopia (2)	0	36	0	10	0	6	0	0
Ghana (2)	0	12	0	0	0	0	0	0
Guinea (2)	0	44	0	6	0	6	0	0
Guinea-Bissau (2)	0	0	0	3	0	0	0	0
Israel (3)	0	0	0	0	0	0	0	1
Liberia (2)	0	0	0	3	0	2	0	0
Madagascar (1)	0	2	0	13	0	5	0	1
Malaysia (1)	0	1	0	0	0	0	0	0
Mali (2)	0	52	0	0	0	0	0	0
Mozambique (2)	0	0	0	2	0	1	0	0
Niger (2)	0	10	0	17	0	0	0	0
Nigeria (2)	0	8	0	415	0	14	0	20
Philippines (2)	0	1	0	0	0	0	0	0
Republic of the Congo (2)	0	2	0	2	0	2	0	0
Senegal (2)	0	0	0	17	0	7	0	0
Sierra Leone (2)	0	10	0	5	0	5	0	0
Somalia (2)	0	14	0	1	0	0	0	2
South Sudan (2)	0	50	0	9	0	9	0	0
Sudan (2)	0	59	0	0	0	0	0	0
Tajikistan (2)	0	1	0	32	0	14	0	0
Togo (2)	0	9	0	0	0	0	0	0
Ukraine (2)	0	0	0	2	0	0	0	0
Yemen (1,2)	0	31	0	64	0	3	0	5
**Total**	**140**	**1,113**	**6**	**688**	**2**	**135**	**3**	**55**

**Abbreviations:** cVDPV = circulating vaccine-derived poliovirus; WPV1 = wild poliovirus type 1.

* Data as of May 5, 2022

WPV1 was detected in specimens from a girl aged 3.5 years living in Lilongwe, Malawi, who had paralysis onset in November 2021. WPV1 was confirmed in February 2022. Genomic sequencing analysis showed the strain detected in Malawi was genetically linked to poliovirus circulating in Sindh province, Pakistan during 2019–2020. On the basis of the number of nucleotide changes from the closest type 1 virus from Pakistan, the Malawi strain was assumed to have circulated in unknown locations for approximately 18 months before its detection.

**Countries reporting cVDPV cases and isolations.** During January 2020–April 2022, a total of 1,856 cVDPV cases were identified in 33 countries. Three countries reported 51 cVDPV1 cases, and 30 countries reported 1,804 cVDPV2 cases. Israel reported one cVDPV3 case and one country, Yemen, reported cases of both cVDPV1 and cVDPV2. The number of global cVDPV2 cases fell by 37.7% in 2021 (672 cases in 21 countries) compared with 2020 (1,079 cases in 24 countries) (Table 2). Thirty-four different active poliovirus emergence groups (lineages) were reported through AFP surveillance or ES in 2021: four cVDPV1, 27 cVDPV2, and three cVDPV3. Of the 27 cVDPV2 emergence groups reported in 2021, eight were newly detected emergences. In 2022, to date, isolations of 14 cVDPV emergence groups have been reported from all three serotypes.

## Discussion

After the last identified indigenous WPV1 case in Nigeria in 2016, the WHO African Region was certified WPV-free in August 2020. In 2021, the region reported its first case of WPV1 in approximately 5 years. In the absence of sustained transmission, this single case does not change the Africa Region’s WPV-free status. Afghanistan and Pakistan continue to have endemic WPV1 circulation; thus, only one WHO region (the Eastern Mediterranean Region) is not certified WPV-free. Although substantial improvements in eradication activities have been made in both countries, insecurity, instability, mass population movements, and vaccine refusal continue to pose challenges. COVID-19 pandemic prevention efforts have affected AFP surveillance sensitivity and the administration of routine childhood immunizations globally (*4*,*7*–*9*). Despite these setbacks, a marked reduction in WPV1 transmission occurred in 2021, possibly linked to improvements in SIA quality, decreased population movement at the start of the COVID-19 pandemic, and renewed national commitments to the program (*8*). While recovery of sensitive AFP surveillance has been limited in 2021 and 2022, the observed reduction in the proportion of WPV1-positive ES samples reported during this period is consistent with a genuine decline in poliovirus transmission.

In Afghanistan, restrictions on house-to-house vaccination campaigns that have been in place in many areas since 2018 have further limited eradication progress. After the shift in political power in Afghanistan in August 2021, mosque-to-mosque polio vaccination campaigns resumed in certain regions of the country, reaching approximately 2 million children who had not been accessible for nearly 3 years, and a coordinated campaign with Pakistan took place in December 2021 (*9*). If these vaccination efforts continue and are extended to include house-to-house campaigns, additional progress toward interrupting WPV1 transmission is feasible during 2022–2023.

To end cVDPV2 transmission by the end of 2023, the 2022–2026 GPEI Strategic Plan (*4*) aims to improve the timeliness of case detection, streamline emergency response structures, and improve cross-border coordination to facilitate prompt outbreak response mobilization. The plan also aims to support the scale-up of nOPV2 availability (*6*). However, given currently limited nOPV2 supply replenishment, higher than expected demand has depleted nOPV2 stock (*3*,*4*). The risk for international spread of polioviruses was declared a Public Health Emergency of International Concern in 2014; in 2021, the Strategic Advisory Group of Experts on Immunization and other advisory bodies^†††^^,^^§§§^ recommended that any country experiencing a cVDPV2 outbreak should begin prompt outbreak response with available OPV2 vaccine, whether it be a Sabin strain mOPV2 or nOPV2 (*10*).

Current progress toward polio eradication needs to be sustained in countries experiencing endemic transmission and outbreaks, and multiple efforts to immunize all children must be enhanced. Ongoing circulation of WPV1 in Afghanistan and Pakistan in 2022 continues to pose a risk for poliovirus exportation globally, further highlighted by detection of WPV1 from Malawi genetically linked to the region. Until WPV1 is eradicated and cVDPV transmission is interrupted, the risk for poliovirus exportation to polio-free areas of the world remains. Strong global efforts are needed to sustain and increase routine immunization coverage and maintain sensitive poliovirus surveillance.

**Summary**

**What is already known about this topic?**

Wild poliovirus type 1 (WPV1) transmission remains endemic in Afghanistan and Pakistan. Outbreaks of paralysis due to circulating vaccine-derived polioviruses (cVDPVs) occur in populations with low immunity following prolonged circulation of Sabin strain oral poliovirus vaccine.

**What is added by this report?**

In 2021, Afghanistan and Pakistan reported a sharp decline in WPV1 cases from previous years. A WPV1 case genetically linked to these countries occurred in Malawi in November 2021.

**What are the implications for public health practice?**

Current progress toward polio eradication must be sustained in countries experiencing endemic transmission and outbreaks. Intensified programmatic actions leading to more effective outbreak responses and enhanced efforts to immunize all children are essential. Until WPV1 is eradicated and cVDPV transmission is interrupted, the risk for children being paralyzed by polio remains.

## Footnotes

* 45 C.F.R. part 46.102(l)(2), 21 C.F.R. part 56; 42 U.S.C. Sect. 241(d); 5 U.S.C. Sect. 552a; 44 U.S.C. Sect. 3501 et seq.

† 2020 is the most recent year for which data are available.

§
https://cdn.who.int/media/docs/default-source/immunization/tables/fipv-considerations-for-decision-making-april2017.pdf?sfvrsn=cff574f9_4

¶ SIAs are mass immunization campaigns intended to interrupt poliovirus circulation by vaccinating every child aged <5 years with 2 OPV doses, irrespective of previous immunization status.

** Emergency Use Listing Procedure is a risk-based procedure for assessing and listing unlicensed vaccines, therapeutics, and in vitro diagnostics with the ultimate aim of expediting the availability of these products to persons affected by a public health emergency. https://www.who.int/teams/regulation-prequalification/eul

†† NPAFP rate is the number of NPAFP cases per 100,000 children aged <15 years per year. An NPAFP rate ≥2 is considered sufficiently sensitive to detect circulating poliovirus.

§§ Stool adequacy is defined as having two stool specimens collected ≥24 hours apart and within 14 days of paralysis onset, and arrival at a WHO-accredited laboratory by reverse cold chain (storing and transporting samples at recommended temperatures from the point of collection to the laboratory) and in good condition (i.e., without leakage or desiccation). Stool specimens are tested using cell culture methods, and any polioviruses isolated have serotype and intratype determined. https://polioeradication.org/wp-content/uploads/2017/05/Polio_Lab_Manual04.pdf

¶¶ 2021 priority countries (2022–2024 Global Polio Surveillance Action Plan priority countries are indicated by [G]). *African Region*: Angola (G), Benin (G), Burkina Faso (G), Cameroon (G), Central African Republic (G), Chad (G), Côte d’Ivoire (G), Democratic Republic of the Congo (G), Equatorial Guinea (G), Ethiopia (G), Gambia, Guinea (G), Guinea-Bissau (G), Kenya (G), Liberia, Madagascar (G), Malawi, Mali (G), Mauritania, Mozambique (G), Niger (G), Nigeria (G), Republic of the Congo, Senegal, Sierra Leone, South Sudan (G), Togo (G), and Uganda; *Eastern Mediterranean Region*: Afghanistan (G), Djibouti, Egypt, Iran, Iraq (G), Pakistan (G), Somalia (G), Sudan (G), Syria (G), and Yemen (G); *European Region*: Tajikistan and Ukraine; *South-East Asia Region*: Burma (Myanmar) (G); *Western Pacific Region*: Papua New Guinea (G) and Philippines (G).

***
https://polioeradication.org/where-we-work/pakistan/

††† WHO Director-General’s Emergency Committee for the International Health Regulations. http://apps.who.int/iris/bitstream/handle/10665/341623/WER9622-eng-fre.pdf

§§§ GPEI Independent Monitoring Board. https://polioeradication.org/wp-content/uploads/2021/07/20th-IMB-report-20210631.pdf
